# Further contributions to the longhorn beetle (Coleoptera, Cerambycidae) fauna of New Brunswick and Nova Scotia, Canada

**DOI:** 10.3897/zookeys.552.6039

**Published:** 2016-01-13

**Authors:** Reginald P. Webster, Chantelle A. Alderson, Vincent L. Webster, CoryC. Hughes, Jon D. Sweeney

**Affiliations:** 1 24 Mill Stream Drive, Charters Settlement, NB, Canada E3C 1X1; 2 Natural Resources Canada, Canadian Forest Service - Atlantic Forestry Centre, 1350 Regent St., P.O. Box 4000, Fredericton, NB, Canada E3B 5P7

**Keywords:** Cerambycidae, new records, Canada, New Brunswick, Nova Scotia

## Abstract

Sixteen species of Cerambycidae are newly recorded for New Brunswick, Canada; *Arhopalus obsoletus* (Randall), *Atimia confusa confusa* (Say), *Callidium frigidum* Casey, *Phymatodes amoenus* (Say), *Phymatodes testaceus* (Linnaeus), *Neoclytus mucronatus mucronatus* (Fabricius), *Xylotrechus aceris* Fisher, *Xylotrechus sagittatus sagittatus* (Germar), *Tylonotus bimaculatus* Haldeman, *Lepturges angulatus* (LeConte), *Lepturges symmetricus* (Haldeman), *Urgleptes querci* (Fitch), *Oplosia nubila* (LeConte), *Eupogonius subarmatus* (LeConte), *Monochamus carolinensis* (Olivier), and *Pogonocherus parvulus* LeConte. *Urgleptes signatus* (LeConte) and *Urgleptes querci* are newly recorded from Nova Scotia. All but two specimens were collected in 12-funnel Lindgren traps. *Xylotrechus aceris*, *Tylonotus bimaculatus*, *Lepturges angulatus*, *Lepturges symmetricus*, *Urgleptes signatus* (NS), and *Pogonocherus parvulus* were detected exclusively in traps deployed in the forest canopy, and most individuals of *Oplosia nubila* and *Monochamus carolinensis* were captured in canopy traps. *Arhopalus obsoletus*, *Atimia confusa confusa*, *Callidium frigidum*, *Phymatodes testaceus*, and *Xylotrechus sagittatus sagittatus* were captured almost exclusively in traps near (1 m above) the forest floor. These results highlight the importance of sampling both the understory and upper canopy when using traps for surveying diversity of Cerambycidae.

## Introduction

The Cerambycidae (longhorn beetles) fauna of New Brunswick was first reviewed by Webster et al. (2009). In that review, 48 species were reported as new to the province. Majka et al. (2010) added two more species to the faunal list. Most recently, Webster et al. (2012a) added six more species and removed *Phymatodes testaceus* (Linnaeus) from the faunal list. Additional species of Cerambycidae were newly recorded from New Brunswick and Nova Scotia during a study to develop tools for enhanced detection of invasive species of Cerambycidae. The purpose of this paper is to report these new records.

## Methods and conventions

### Collection methods.

All specimens but two (which were hand collected) were collected from Lindgren 12-funnel trap samples during studies to develop improved tools for detection of invasive species of Cerambycidae. Lindgren funnel traps are visually similar to tree trunks and are often effective for sampling species of Coleoptera that live in microhabitats associated with standing trees (Lindgren 1983). The species records come from samples collected in more than 800 funnel traps deployed at 17 sites (24–64 traps per site) between 2012 and 2015. At most sites, equal numbers of traps were deployed in the upper canopy as well as in the understory, but three sites were sampled only with canopy traps, and five sites were sampled only with understory traps. Overall, understory traps outnumbered canopy traps by a factor of 1.3. Canopy traps were 10–20 m above the ground, whereas understory traps were 1–1.5 m above the ground (i.e., 30–50 cm from the bottom of the collecting cup to the ground). In both cases, traps were suspended from rope such that the trap was at least 1 m from the main stem of trees and at least 30 m from another trap. For details of the methods used to deploy Lindgren traps and for sample collection, please see Webster et al. (2012b) and Hughes et al. (2014).

Traps were baited with various multi-lure combinations of plant volatiles and longhorn beetle aggregation/sex pheromones that varied among sites and years. These included high release-rate lures of ethanol and alpha-pinene, as well as hydroxyketones, hexanediols, 2-undecyloxy-1-ethanol (commonly known as monochamol), ipsenol, (*E*,*Z*)-6,10-dimethyl-5,9-undecadien-2-ol [(*E*,*Z*)-fuscumol] and (*E*,*Z*)-6,10-dimethyl-5, 9-undecadien-2-yl acetate [(*E*,*Z*)-fuscumol acetate]. The fuscumol and fuscumol acetate lures were purchased from Sylvar Technologies (Fredericton, NB). The hexanediols were synthesized at Atlantic Forestry Centre, and the hydroxyketones were purchased from Bedoukian Research (Danbury, CT), and both were loaded into release devices at Contech Enterprises Inc. (Delta, BC). All other lures were purchased directly from Contech Enterprises Inc. (Delta, BC). Traps baited with these pheromones and plant volatiles have been shown to increase trap catches of many species of longhorn beetles (Lacey et al. 2004, 2009, Hanks et al. 2007, Hanks and Millar 2013, Silk et al. 2007, Pajares et al. 2010, Allison et al. 2012, Ryall et al. 2014, Sweeney et al. 2014).

A description of the habitat was recorded for all specimens collected during this survey. Locality and habitat data are presented as on labels for each record. Two labels were used on many specimens, one that included the locality, collection date, and collector, and one with macro- and micro-habitat data and collection method. Information from the two labels is separated by a // in the data presented from each specimen. This information, as well as additional published data, is summarized and discussed in the collection and habitat data section for each species.

### Distribution.

Every species is cited with current distribution in Canada and Alaska, using abbreviations for the state, provinces, and territories. New records for New Brunswick are indicated in **bold** under

### Distribution in Canada and Alaska.

The following abbreviations are used in the text:

AK
 Alaska 


MB
 Manitoba 


YT
 Yukon Territory 


ON
 Ontario 


NT
 Northwest Territories 


QC
 Quebec 


NU
 Nunavut 


NB
 New Brunswick 


BC
 British Columbia 


PE
 Prince Edward Island 


AB
 Alberta 


NS
 Nova Scotia 


SK
 Saskatchewan 


NF & LB
 Newfoundland and Labrador *

*Newfoundland and Labrador are each treated separately under the current distribution in Canada and Alaska.

Acronyms of collections examined or where specimens reside referred to in this study are as follows:

AFC
 Atlantic Forestry Centre, Fredericton, New Brunswick, Canada 


CNC
 Canadian National Collection of Insects, Arachnids and Nematodes, Ottawa, Ontario, Canada 


KNPC
 Kouchibouguac National Park Collection, New Brunswick, Canada 


NBM
 New Brunswick Museum, Saint John, New Brunswick, Canada 


RWC
 Reginald P. Webster Collection, Charters Settlement, New Brunswick, Canada 


## Results

### Species accounts

All records below are species newly recorded for New Brunswick or Nova Scotia, Canada. The determination that a species was a new record was based on information in the print version of Bousquet et al. (2013). Species designated with a † are adventive to Canada.

### Family Cerambycidae Latreille, 1802

#### Subfamily Spondylidinae Audinet-Serville, 1832

##### Tribe Asemini J. Thomson, 1860

###### Arhopalus obsoletus

Taxon classificationAnimaliaColeopteraCerambycidae

(Randall, 1838)

####### Material examined.

**New Brunswick, Northumberland Co.**, ca. 2.5 km W of Sevogle, 47.0876°N, 65.8613°W, 8–22.VII.2013, 22.VII-6.VIII.2013, 9–23.VII.2014, C. Alderson & V. Webster // Old *Pinus banksiana* stand, Lindgren funnel traps (3, AFC; 2, RWC).

####### Collection and habitat data.

Adults were captured in Lindgren funnel traps in an old jack pine (*Pinus banksiana* Lamb.) forest during July and August. Larvae feed at the base and in roots of dead pines (Yanega 1996).

####### Distribution in Canada and Alaska.

ON, **NB** (Bousquet et al. 2013).

##### Tribe Atimiini LeConte, 1873

###### Atimia confusa confusa

Taxon classificationAnimaliaColeopteraCerambycidae

(Say, 1826)

####### Material examined.

**New Brunswick, York Co.**, Canterbury, Eel River P.N.A. (Protected Natural Area), 45.8967°N, 67.6343°W, 21.V–2.VI.2014, 2–20.VI.2014, 25.VIII-2.IX.2014, C. Alderson & V. Webster // Old-growth eastern white cedar swamp & fen, Lindgren funnel traps (2, AFC; 2, RWC); Keswick Ridge, 45.9962°N, 66.8781°W, 22.V-4.VI.2014, C. Alderson & V. Webster // Mixed forest, Lindgren funnel trap in canopy (1, AFC).

####### Collection and habitat data.

Specimens of *Atimia confusa confusa* were caught in Lindgren traps in an old-growth eastern white cedar (*Thuja occidentalis* L.) swamp and fen during May, June, August, and September. Most individuals were caught in traps in the open part of the fen. One individual was captured in a Lindgren trap deployed in the canopy of a tree in a mixed forest with eastern white cedar. Yanega (1996) states that larvae of this species develop under bark of cedars and junipers (*Juniperus* sp.), and cypresses (*Taxodium* sp.) but does not give any details on the species.

####### Distribution in Canada and Alaska.

ON, QC, **NB** (Bousquet et al. 2013).

#### Subfamily Cerambycinae Latreille, 1802

##### Tribe Callidiini Kirby, 1837

###### Callidium frigidum

Taxon classificationAnimaliaColeopteraCerambycidae

Casey, 1912

####### Material examined.

**New Brunswick, York Co.**, Douglas, Currie Mountain, 45.9832°N, 66.7564°W, 27.V-10.VI.2013, C. Alderson & V. Webster // Old *Pinus strobus* stand, Lindgren funnel trap in canopy of *Pinus strobus* (1, AFC); Canterbury, Eel River P.N.A., 45.8967°N, 67.6343°W, 2–20.VI.2014, C. Alderson & V. Webster // Old-growth eastern white cedar swamp & fen, Lindgren funnel traps (6, AFC; 1, NBM; 5, RWC).

####### Collection and habitat data.

Specimens of *Callidium frigidum* were captured in Lindgren traps in an old-growth eastern white cedar swamp and fen during June. One individual was caught in a Lindgren funnel trap in the canopy of a stand of white pine (*Pinus strobus* L.) with scattered eastern white cedar. Larvae develop under bark of juniper and cedar (Yanega 1996).

####### Distribution in Canada and Alaska.

ON, QC, **NB**, NF (Bousquet et al. 2013).

###### Phymatodes amoenus

Taxon classificationAnimaliaColeopteraCerambycidae

(Say, 1824)

####### Material examined.

**New Brunswick, York Co.**, Keswick Ridge, 45.9962°N, 66.8781°W, 3–18.VI.2015, 20.VI-16.VII.2015, C. Alderson & V. Webster // Mixed forest, Lindgren funnel trap in canopy (4), 1 m high under trees (2) (3, AFC; 3, RWC); same locality and collectors but 3–18.VI.2015, 18–30.VI.2015 // Hardwood forest, green Lindgren funnel trap in canopy (2), purple Lindgren trap in canopy (1), green Lindgren trap 1 m high under trees (2) (2, AFC; 3, RWC).

####### Collection and habitat data.

*Phymatodes amoenus* was captured in Lindgren traps on the edge of a mixed forest and edge of nearby hardwood stand adjacent to a field. Seven of the 11 individuals were captured in traps in the canopy of trees. Larvae of this species mine under bark of dead grapevines (Yanega 1996). Our only native grape, *Vitis labrusca* L. occurred at several areas along the margin of the mixed and hardwood forest where traps were deployed and is the presumed host in New Brunswick.

####### Distribution in Canada and Alaska.

ON, QC, **NB** (Bousquet et al. 2013).

###### Phymatodes testaceus

Taxon classificationAnimaliaColeopteraCerambycidae

(Linnaeus, 1758)†

####### Material examined.

**New Brunswick, York Co.**, Fredericton, Odell Park, 45.9571°N, 66.6650°W, 1–15.VI.2012, 15–28.VI.2012, 10–26.VII.2012, C. Alderson & V. Webster // Old-growth eastern hemlock forest, Lindgren funnel traps 1 m high under *Betula alleghaniensis* (2, AFC; 2, RWC); same locality and collectors but 45.9484°N, 66.6802°W, 17.VI-3.VII.2014 // Old mixed forest, Lindgren funnel trap 1 m high under trees (1, AFC; 1, RWC).

####### Collection and habitat data.

Adults of this introduced species were caught during June and July in Lindgren funnel traps in an urban park with sections of old-growth eastern hemlock (*Tsuga canadensis* (L.) Carr.) and mixed forest. All adults were caught in traps in the understory. The immature stages of this species develop under bark of various hardwoods and pine (Yanega 1996).

####### Distribution in Canada and Alaska.

BC, ON, QC, **NB**, NS (Bousquet et al. 2013).

####### Comments.

Webster et al. (2009) reported *Phymatodes testaceus* from New Brunswick based on a series of specimens from Pleasantfield. It was determined that these specimens were mislabeled and were from Pleasantfield, Nova Scotia. Webster et al. (2012a) accordingly removed the species from the faunal list of New Brunswick. This species is reinstated to the faunal list of New Brunswick based on the above records.

##### Tribe Clytini Mulsant, 1839

###### Neoclytus mucronatus mucronatus

Taxon classificationAnimaliaColeopteraCerambycidae

(Fabricius, 1775)

####### Material examined.

**New Brunswick, York Co.**, 16 km W of Tracy off Rt. 645, 45.6854°N, 66.8839°W, 11–25.VII.2014, C. Alderson & V. Webster // Old red pine forest, Lindgren funnel trap (1, AFC: 1, RWC).

####### Collection and habitat data.

Two specimens of *Neoclytus mucronatus mucronatus* were captured during July in a Lindgren funnel trap baited with a multi-lure combination that included its aggregation pheromone, 3-hydroxyhexan-2-one, placed in the understory of an old red pine (*Pinus resinosa* Ait.) forest. According to Yanega (1996), larvae of this species develop under bark of dead and dying hickory (*Carya* sp.) (which does not occur in New Brunswick) and rarely pine. Presence of the hydroxyketone lure is likely responsible for detecting *Neoclytus mucronatus mucronatus* because it contains the aggregation pheromone identified for this species (Lacey et al. 2007). Failure to detect *Neoclytus mucronatus mucronatus* in traps baited with its aggregation pheromone at 13 other sites in New Brunswick from 2012–2014 suggests its occurrence in New Brunswick is rare or localized.

####### Distribution in Canada and Alaska.

ON, **NB** (Bousquet et al. 2013).

###### Xylotrechus aceris

Taxon classificationAnimaliaColeopteraCerambycidae

Fisher, 1917

####### Material examined.

**New Brunswick, Carleton Co.**, Jackson Falls, “Bell Forest”, 46.2200°N, 67.7231°W, 31.VII-14.VIII.2012, C. Alderson & V. Webster // Rich Appalachian hardwood forest, Lindgren funnel traps in canopy of *Acer saccharum* (1, AFC; 1, RWC).

####### Collection and habitat data.

Both adults were caught in Lindgren funnel traps in the canopy of sugar maples (*Acer saccharum* Marsh.) in a hardwood forest during August. The larvae develop in branches of live maple (*Acer* sp.) (Yanega 1996).

####### Distribution in Canada and Alaska.

ON, QC, **NB** (Bousquet et al. 2013).

###### Xylotrechus sagittatus sagittatus

Taxon classificationAnimaliaColeopteraCerambycidae

(Germar, 1821)

####### Material examined.

**New Brunswick, Kent Co.**, Kouchibouguac National Park, 46.816821°N, 64.915475°W, 23.VIII.2012 // Salt marsh, flight intercept trap baited with spruce blend, ethanol, fuscumol (1, KNPC); same locality but 46.8072°N, 64.9100°W, 4–20.VIII.2015, 20–31.VIII.2015, C. Alderson & V. Webster // Jackpine forest, Lindgren funnel traps, 1 m high (5, AFC; 2, RWC). **Northumberland Co.**, ca. 2.5 km W of Sevogle, 47.0876°N, 65.8613°W, 8–21.VIII.2013, 23.VII-6.VIII.2014, 6–20.VIII.2014, 20.VIII-3.IX.2014, C. Alderson & V. Webster // Old *Pinus banksiana* stand, Lindgren funnel traps (5, AFC; 1, NBM; 5, RWC). **Queens Co.**, C.F.B. Gagetown, 45.7516°N, 66.1866°W, 15–31.VII.2013, C. Alderson & V. Webster // Old mixed forest with *Quercus rubra*, Lindgren funnel trap in canopy of *Quercus rubra* (1, AFC).

####### Collection and habitat data.

Most individuals in New Brunswick were caught in Lindgren funnel traps in old jack pine forests. One adult was captured in a Lindgren funnel trap in the canopy of a red oak (*Quercus rubra* L.) adjacent to a white pine stand; another from a flight intercept trap in a salt marsh next to a jack pine stand. Yanega (1996) reports *Pinus* as the main larval host of this species. Adults were captured during July, August, and September.

####### Distribution in Canada and Alaska.

MB, ON, QC, **NB**, NS, PE (Bousquet et al. 2013).

##### Tribe Hesperophanini Mulsant, 1839

###### Tylonotus bimaculatus

Taxon classificationAnimaliaColeopteraCerambycidae

Haldeman, 1847

####### Material examined.

**New Brunswick, Queens Co.**, C.F.B. Gagetown, 45.7516°N, 66.1866°W, 15–31.VII.2013, C. Alderson & V. Webster // Old mixed forest with *Quercus rubra*, Lindgren funnel trap in canopy of *Quercus rubra* (1, AFC). **Sunbury Co.**, Gilbert Island, 45.8770°N, 66.2954°W, 25.VII-8.VIII.2012, 5–17.VII.2013, C. Alderson, C. Hughes, & V. Webster // Hardwood forest, Lindgren funnel trap in canopy of *Tilia americana* (1), and canopy of *Fraxinus pennsylvanica* (2) (1, AFC; 2, RWC).

####### Collection and habitat data.

All specimens (4) of *Tylonotus bimaculatus* from New Brunswick were captured in Lindgren funnel traps in the canopy of trees (red oak, basswood (*Tilia americana* L.), green ash (*Fraxinus pennsylvanica* Marsh.)) in mixed and hardwood forests with ash (*Fraxinus* sp.). Hosts include live or dying hardwoods, especially ash (Yanega 1996). Adults were captured during July and August.

####### Distribution in Canada and Alaska.

MB, ON, QC, **NB** (Bousquet et al. 2013).

#### Subfamily Lamiinae Latreille, 1825

##### Tribe Acanthocinini Blanchard, 1845

###### Lepturges angulatus

Taxon classificationAnimaliaColeopteraCerambycidae

(LeConte, 1852)

####### Material examined.

**New Brunswick, Northumberland Co.**, Upper Graham Plains, 47.1001°N, 66.8154°W, 24.VII-7.VIII.2014, C. Alderson & V. Webster // Old black spruce (*Picea mariana* (Mill.) B.S.P.) forest with white pine, Lindgren funnel trap in canopy of white pine (1, AFC). **York Co.**, Fredericton, Odell Park, 45.9484°N, 66.6802°W, 1–15.VIII.2014, C. Alderson & V. Webster // Old mixed forest, Lindgren funnel traps in canopy of hardwoods (1, AFC; 1, RWC).

####### Collection and habitat data.

All adults (3) of *Lepturges angulatus* from New Brunswick were captured in Lindgren funnel traps in the canopy of trees; one from the canopy of a white pine in an old black spruce forest with white pine and two from the canopy of hardwoods in an old mixed forest stand. Yanega (1996) reports various hardwoods and pine as larval hosts of this species. Adults were collected during July and August in New Brunswick.

####### Distribution in Canada and Alaska.

ON, QC, **NB** (Bousquet et al. 2013).

###### Lepturges symmetricus

Taxon classificationAnimaliaColeopteraCerambycidae

(Haldeman, 1847)

####### Material examined.

**New Brunswick, Carleton Co.**, Jackson Falls, “Bell Forest”, 46.2200°N, 67.7231°W, 21.VI-3.VII.2012, C. Alderson & V. Webster // Rich Appalachian hardwood forest, Lindgren funnel trap in canopy of *Tilia americana* (1, AFC). **Sunbury Co.**, Gilbert Island, 45.8770°N, 66.2954°W, 12–29.VI.2012, C. Alderson, C. Hughes, & V. Webster // hardwood forest, Lindgren funnel trap in canopy of *Juglans cinerea* (1, RWC). **York Co.**, Fredericton, Odell Park, 45.9539°N, 66.6666°W, 9–24.VII.2013, C. Alderson & V. Webster // Hardwood stand, Lindgren funnel trap in canopy (1, AFC); Keswick Ridge, 45.9962°N, 66.8781°W, 13–27.VIII.2015, C. Alderson & V. Webster // Mixed forest, Lindgren funnel trap in canopy (1, RWC).

####### Collection and habitat data.

All adults (4) of *Lepturges symmetricus* from New Brunswick were captured in Lindgren funnel traps in the canopy of trees (American beech (*Fagus grandifolia* Ehrh.), butternut (*Juglans cinerea* L.)) in hardwood and mixed forests. Larval hosts include branches of various hardwoods (Yanega 1996). Adults were captured during June, July, and August.

####### Distribution in Canada and Alaska.

ON, QC, **NB** (Bousquet et al. 2013).

###### Urgleptes querci

Taxon classificationAnimaliaColeopteraCerambycidae

(Fitch, 1858)

####### Material examined.

**New Brunswick, Carleton Co.**, Jackson Falls, “Bell Forest”, 46.2200°N, 67.7231°W, 3–17.VII.2012, 31.VII-14.VIII.2012, C. Alderson & V. Webster // Rich Appalachian hardwood forest, Lindgren funnel traps in canopy of *Acer saccharum* (1), *Fagus grandifolia* (1), and *Juglans cinerea* (1) (1, AFC; 2, RWC). **Restigouche Co.**, Jacquet River Gorge P.N.A., 47.8257°N, 66.0764°W, 5–19.VIII.2014, C. Alderson & V. Webster // Old *Populus balsamifera* stand near river, Lindgren funnel trap 1 m high under trees (1, NBM); ca. 3 km SE of Simpsons Field, 47.5277°N, 66.5142°W, 23.VI-5.VIII.2015, C. Alderson & V. Webster // Old cedar & spruce forest with *Populus balsamifera* & *Populus tremuloides*, Lindgren funnel trap (1, AFC). **Sunbury Co.**, Gilbert Island, 45.8770°N, 66.2954°W, 29.VI-11.VII.2012, 25.VII-8.VIII.2012, 8–21.VIII.2012, C. Alderson, C. Hughes, & V. Webster // hardwood forest, Lindgren funnel traps 1 m high under *Juglans cinerea* (8), in canopy of *Juglans cinerea* (3), and 1 m high under *Tilia americana* (5) (9, AFC; 1, NBM; 6, RWC): same data as previous record, but 5–17.VII.2013, Lindgren funnel trap in canopy of *Ulmus americana* (1, AFC). **Victoria Co.**, Saint Leonard, mesotrophic sugar maple forest, 16.VII.2014, John Klymko (1, NBM). **York Co.**, Douglas, Currie Mountain, 45.9844°N, 66.7592°W, 24.VII-7.VIII.2013, 6–17.IX.2013, C. Alderson & V. Webster // Mixed forest with *Quercus rubra*, Lindgren funnel traps 1 m high under *Quercus rubra* (2 AFC); Fredericton, Odell Park, 45.9539°N, 66.6666°W, 24.VI-9.VII.2013, 7–19.VIII.2013, C. Alderson & V. Webster // Hardwood stand, Lindgren funnel traps in canopy (2), Lindgren funnel trap 1 m under trees (1) (3, AFC); Keswick Ridge, 45.9962°N, 66.8781°W, 3–18.VII.2014, C. Alderson & V. Webster // Mixed forest, Lindgren funnel trap in canopy (1, RWC); Canterbury, Eel River P.N.A., 45.8966°N, 67.6345°W, 15–28.VII.2014, C. Alderson & V. Webster // Old-growth eastern white cedar swamp & fen, Lindgren funnel trap (1, NBM). **Nova Scotia, Halifax Co.**, Magazine Hill, 44°42'19.1"N, 63°37"19.89"W, 11.VIII.2014, Sweeney Lab, coll. // High-Low Experiment, Ketols Lure, High Traps (2, AFC).

####### Collection and habitat data.

In New Brunswick, most *Urgleptes querci* adults were captured in Lindgren funnel traps in hardwood and mixed forests; one individual was caught in a Lindgren trap in an old-growth eastern white cedar swamp, and another was hand-collected in a sugar maple forest. The two adults from Nova Scotia were captured in the canopy of trees in a mixed forest. Larvae of this species develop in branches of many hardwoods, especially maple, shrubs, and vines (Yanega 1996). This species was collected from June into September.

####### Distribution in Canada and Alaska.

ON, QC, **NB**, **NS** (Bousquet et al. 2013).

###### Urgleptes signatus

Taxon classificationAnimaliaColeopteraCerambycidae

(LeConte, 1852)

####### Material examined.

**Nova Scotia, Halifax Co.**, Magazine Hill, 44°, 42’, 19.1”N, 63°, 37”, 19.89”W, 21.VII.2014, 28.VII.2014, 4.VIII.2014, Sweeney Lab, coll. // High-Low Experiment, Mono Lure, High Trap (1), Mono Lure, Low Trap (1), Ketols Lure, High Trap (1) (3, AFC).

####### Collection and habitat data.

Two of the three specimens from Nova Scotia were captured in Lindgren funnel traps in the canopy of trees in a mixed forest.

####### Distribution in Canada and Alaska.

ON, QC, NB, **NS** (Bousquet et al. 2013).

##### Tribe Acanthoderini J. Thomson, 1860

###### Oplosia nubila

Taxon classificationAnimaliaColeopteraCerambycidae

(LeConte, 1862)

####### Material examined.

**New Brunswick, Carleton Co.**, Jackson Falls, “Bell Forest”, 46.2200°N, 67.7231°W, 21.VI-3.VII.2012, C. Alderson & V. Webster // Rich Appalachian hardwood forest, Lindgren funnel trap in canopy of *Tilia americana* (1, RWC). **Sunbury Co.**, Gilbert Island, 45.8770°N, 66.2954°W, 12–29.VI.2012, 29.VI-11.VII.2012, 11–25.VII.2012, C. Alderson, C. Hughes, & V. Webster // hardwood forest, Lindgren funnel traps 1 m high under *Tilia americana* (9) and in canopy of *Tilia americana* (13) (11, AFC; 1, CNC; 1, NBM; 9, RWC); same data as previous record but 5–17.VII.2013, Lindgren funnel trap in canopy of *Ulmus americana* (1, AFC). **York Co.**, Fredericton, Odell Park, 45.9539°N, 66.6666°W, 10–24.VI.2013, 9–24.VII.2013, C. Alderson & V. Webster // Hardwood stand, Lindgren funnel traps in canopy of *Fraxinus americana* L. and *Fagus grandifolia* Ehrh. (2, AFC); Keswick Ridge, 45.9962°N, 66.8781°W, 3–18.VII.2014, C. Alderson & V. Webster // Mixed forest, Lindgren funnel trap in canopy (1, AFC).

####### Collection and habitat data.

Most (17 out of 27) individuals of *Oplosia nubila* from New Brunswick were captured in Lindgren funnel traps in the canopy of trees. Larvae of this species develop under bark of decaying basswood, hickory (which does not occur in New Brunswick), and beech (Yanega 1996). In New Brunswick, nearly all adults were captured in traps that were either in the canopy or understory of basswood trees at sites where other tree species were also sampled.

####### Distribution in Canada and Alaska.

MB, ON, QC, **NB** (Bousquet et al. 2013).

###### Eupogonius subarmatus

Taxon classificationAnimaliaColeopteraCerambycidae

(LeConte, 1859)

####### Material examined.

**New Brunswick, Carleton Co.**, Jackson Falls, “Bell Forest”, 46.2200°N, 67.7231°W, 17.VII.2012, R.P. Webster // Rich Appalachian hardwood forest, on foliage (1, RWC). **York Co.**, Keswick Ridge, 45.9962°N, 66.8781°W, 29.VII-13.VIII.2015, C. Alderson & V. Webster // Mixed forest, Lindgren funnel trap in canopy (1, AFC).

####### Distribution in Canada and Alaska.

ON, QC, **NB** (Bousquet et al. 2013).

###### Monochamus carolinensis

Taxon classificationAnimaliaColeopteraCerambycidae

(Olivier, 1795)

####### Material examined.

**New Brunswick, Kent Co.**, Kouchibouguac National Park, 46.8072°N, 64.9100°W, 7–22.VII.2015, 27.VII-4.VIII.2015, C. Alderson & V. Webster // Jackpine forest, Lindgren funnel traps, 1 m high (5, AFC). **Northumberland Co.**, ca. 2.5 km W of Sevogle, 47.0876°N, 65.8613°W, 8–22.VII.2013, 22.VII-6.VIII.2013, 6–21.VIII.2013, 21.VIII-4.IX.2013, 17.IX-1.X.2013, 1–17.X.2013, C. Alderson & V. Webster // Old *Pinus banksiana* stand, Lindgren funnel traps (10, AFC: 6, CNC; 6, NBM; 3, RWC); Upper Graham Plains, 47.1001°N, 66.8154°W, 9–24.VII.2014, C. Alderson & V. Webster // Old black spruce forest, Lindgren funnel trap (1, AFC). **Queens Co.**, C.F.B. Gagetown, 45.7516°N, 66.1866°W, 2–17.VII.2015, 30.VII-14.VIII.2015, 14–28.VIII.2015, 28.VIII-10.IX.2015, C. Alderson & V. Webster // Old mixed forest with *Quercus rubra*, Lindgren funnel traps in canopy (8, AFC). **Sunbury Co.**, Acadia Research Forest, 45.9990°N, 66.2623°W, 26.VII-7.VIII.2012, 22.VIII-10.IX.2012, C. Hughes & K. Van Rooyen // Mature balsam fir forest with scattered red spruce & red maple (and white pine), Lindgren funnel traps (1, AFC; 1, RWC). **York Co.**, Douglas, Currie Mountain, 45.9832°N, 66.7564°W, 24.VI-9.VII.2013, 9–24.VII.2013, 24.VII-7.VIII.2013, 7–19.VIII.2013, 17.IX-3.X.2013, 3–15.X.2013, C. Alderson & V. Webster // Old *Pinus strobus* stand, Lindgren funnel traps in canopy of *Pinus strobus* (18, AFC; 6, CNC; 17, NBM; 7, RWC); Fredericton, Odell Park, 45.9484°N, 66.6802°W, 3–17.VII.2014, 17.VII-1.VIII.2014, C. Alderson & V. Webster // Old mixed forest, Lindgren funnel trap in canopy of conifer (2), 1 m high under trees (1) (1, AFC; 2, NBM); 16 km W of Tracy, off Rt. 645, 45.6854°N, 66.8839°W, 11–25.VII.2014, 25.VI-8.VII.2014, 18.VIII-5.IX.2014, C. Alderson & V. Webster // Old red pine forest, Lindgren funnel trap in canopy of red pine (3, AFC; 3, NBM).

####### Collection and habitat data.

*Monochamus carolinensis* were captured in Lindgren traps baited with a multi-lure combination that included monochamol, ipsenol, alpha-pinene, and ethanol in a jack pine forest, an old black spruce stand with white pine, a mature balsam fir forest with white pine, an old white pine stand, an old red pine forest, and a mixed forest with red oak and white pine. Hosts include various *Pinus* species according to Yanega (1996). In New Brunswick, large numbers (many specimens were not vouchered) of adults were captured in a white pine stand, mostly in the canopy. This species was also common in a jack pine stand but uncommon in a red pine forest. These data suggest that white pine may be the preferred host in New Brunswick. This species has a long flight season in New Brunswick from early July to mid-October. Ryall et al (2014) showed that catch of *Monochamus carolinensis* was significantly increased by baiting traps with monochamol, ipsenol, alpha-pinene and ethanol.

####### Distribution in Canada and Alaska.

ON, QC, **NB** (Bousquet et al., 2013).

####### Comments.

*Monochamus carolinensis* was listed for New Brunswick by McNamara (1991). Webster et al. (2012a) could not locate any specimens to support its occurrence in the province but included it in the fauna of the province. Bousquet et al. (2013), however, did not list it as occurring in New Brunswick. The above records support reinstatement of the species to the faunal list of New Brunswick.

##### Tribe Pogonocherini Mulsant, 1839

###### Pogonocherus parvulus

Taxon classificationAnimaliaColeopteraCerambycidae

LeConte, 1852

####### Material examined.

**New Brunswick, Gloucester Co.**, Bathurst, Daly Point Nature Preserve, 47.6392°N, 65.6098°W, 25.VI-9.VII.2015, C. Alderson & V. Webster // Mixed forest, black Lindgren trap in canopy (*Populus*)(1, AFC). **Restigouche Co.**, ca. 3 km SE of Simpsons Field, 47.5277°N, 66.5142°W, 25.VI-10.VII.2015, C. Alderson & V. Webster // Old cedar & spruce forest with *Populus balsamifera* & *Populus tremuloides*, Lindgren funnel trap in canopy of *Populus balsamifera* (1, RWC). **Sunbury Co.**, Gilbert Island, 45.8770°N, 66.2954°W, 20.VI-5.VII.2013, C. Alderson, C. Hughes, & V. Webster // hardwood forest, Lindgren funnel traps in canopy of *Acer saccharinum* and *Populus tremuloides* (1, AFC; 1, RWC).

####### Collection and habitat data.

One individual each was captured in Lindgren funnel traps in the canopy of silver maple and trembling aspen (*Populus tremuloides* Michx.) in a hardwood forest on Gilbert Island. At Daly Point and Simpsons Field, *Pogonocherus parvulus* was captured in the canopy of *Populus* sp.. Willow (*Salix*), a common species at the above sites, is listed by Yanega (1996) as a host of this species.

####### Distribution in Canada and Alaska.

AB, SK, MB, ON, QC, **NB** (Bousquet et al. 2013).

## Discussion

These new species records of Cerambycidae for the provinces of New Brunswick and Nova Scotia enrich our knowledge of the region’s fauna and species diversity. With the exception of one specimen of *Eupogonius subarmatus* and one specimen of *Urgleptes querci*, all longhorn beetle specimens were collected in Lindgren multi-funnel traps placed in either the understory or upper canopy. Of the 16 species collected in traps, three species were collected exclusively in understory traps (*Arhopalus obsoletus*, *Atimia confusa confusa*, *Neoclytus mucronatus mucronatus*), and six species were collected exclusively in canopy traps (*Lepturges angulatus*, *Lepturges symmetricus*, *Pogonocherus parvulus*, *Tylonotus bimaculatus*, *Urgleptes signatus*, *Xylotrechus aceris*). These results highlight the need to sample both the canopy and understory when using traps to survey for beetle species. Differences in insect species composition between traps placed in the upper canopy and understory and the importance of sampling both strata have been shown in several forest habitats (Su and Woods 2001; Vance et al. 2003; Graham et al. 2012; Dodds 2014).

## Supplementary Material

XML Treatment for Arhopalus obsoletus

XML Treatment for Atimia confusa confusa

XML Treatment for Callidium frigidum

XML Treatment for Phymatodes amoenus

XML Treatment for Phymatodes testaceus

XML Treatment for Neoclytus mucronatus mucronatus

XML Treatment for Xylotrechus aceris

XML Treatment for Xylotrechus sagittatus sagittatus

XML Treatment for Tylonotus bimaculatus

XML Treatment for Lepturges angulatus

XML Treatment for Lepturges symmetricus

XML Treatment for Urgleptes querci

XML Treatment for Urgleptes signatus

XML Treatment for Oplosia nubila

XML Treatment for Eupogonius subarmatus

XML Treatment for Monochamus carolinensis

XML Treatment for Pogonocherus parvulus
